# Three-Dimensional PrGO-Based Sandwich Composites With MoS_2_ Flowers as Stuffings for Superior Lithium Storage

**DOI:** 10.3389/fchem.2020.00094

**Published:** 2020-02-28

**Authors:** Yangqiang Zhao, Ziying Zhang, Huizhen Zhang, Yangyang Zhou, Ying Weng, Shisheng Xiong

**Affiliations:** ^1^School of Materials Engineering, Shanghai University of Engineering Science, Shanghai, China; ^2^School of Management, University of Shanghai for Science and Technology, Shanghai, China; ^3^School of Information Science and Engineering, Fudan University, Shanghai, China

**Keywords:** molybdenum sulfide, graphene oxide, anode, composites, lithium ion batteries

## Abstract

Graphene-based MoS_2_ nanocomposites are expected to be promising anode materials for lithium ion batteries because of their large specific capacity and high conductivity. However, the aggregation of graphene and the weak interaction between the two components hinder their practical application. Inspired by the sandwich structure, novel three-dimensional flower-like MoS_2_-PrGO sandwich composites were proposed as an advanced anode material for lithium-ion batteries. The separated 2D ultrathin rGO nano-sheets were connected by PEO chains and assembled into a well-organized 3D layered spatial structure, which not only avoids the aggregation of graphene but also accommodates a high mass loading of the micro-scale MoS_2_ nano-flowers. MoS_2_ nano-flowers with open architecture deliver large specific area. The rGO interlayers act as a conductive framework, making all flower-like MoS_2_ nano-stuffing electrochemically active. The ultra-thin 2D nano-sheets provide excellent cycle stability due to their neglectable volume changes during cycling. The 3D flower-like MoS_2_-PrGO sandwich composites deliver high energy density, excellent conductivity and stable cyclic performance during charge-discharge process. With a nearly 100% coulombic efficiency, their reversible capacity is retained at 1,036 mA h g^−1^ even after 500 cycles at current densities of 100 mA g^−1^. This novel design strategy provides a broad prospect for the development of advanced anode materials for superior lithium storage.

## Introduction

Lithium-ion batteries have become the main power source of portable electronic products because of their high energy density, long cycle life, and eco-friendliness (Dunn et al., 2011; Fan et al., 2019; Zhang et al., 2019). With the rapid development of mobile electronic devices and electric vehicles, the market has put forward higher requirements on the energy storage density and stability of batteries. Therefore, more attentions have been paid to the exploration of electrode materials with high capacity and advanced performance (Ding et al., 2019; Zhang et al., 2019).

Transition metal dichalcogenides (TMDs) have become a popular choice in the field of energy storage due to their graphene-like two-dimensional (2D) layered structure, in which lithium ions tend to migrate among the 2D layers due to the weak van der Waals interactions (Yang et al., 2015; Cook et al., 2016; Wang et al., 2016). Among a variety of TMDs, MoS_2_ is an ideal anode material for lithium ion batteries because its proper interlayer spacing endows this material a higher theoretical specific capacity (Kadam et al., 2019; Santhosha et al., 2019). However, its poor conductivity and large volume variations have limited its application as an advanced anode material. To address the above issues, MoS_2_ has been engineered into different nanostructures and nanocomposites (Kadam et al., 2019; Santhosha et al., 2019; Sun et al., 2019; Wang M. et al., 2019; Wang Y. et al., 2019; Xie et al., 2019; Zhu et al., 2019). Recently, due to its excellent electrical, thermal and mechanical properties, graphene has been used to combine with MoS_2_ to form an anode material for lithium-ion batteries (Huang et al., 2017; Tan et al., 2018; Anwer et al., 2019). The high mechanical strength and extraordinary conductivity of graphene greatly improve the structure stability and the electrical performance of the graphene-based MoS_2_ nanocomposites. However, large volume changes occurred when lithium ions were embedded in the MoS_2_ layers and preferentially interacted with S layers to form Li–S bonds. The significant capacity degradation triggered by the changes in MoS_2_ volume still can not be eliminated. Moreover, the solid-electrolyte interphase (SEI) layer produced by organic electrolyte decomposition deforms and fractures during the expansion and contraction of MoS_2_. The new SEI on the newly exposed MoS_2_ surface results in low coulombic efficiency. The gradual accumulation of SEI further impedes the transfer of lithium ions. By self-assembling 2D MoS_2_ nano-sheets into three-dimensional (3D) layered porous nano-spheres with uneven surfaces, the capacity of the MoS_2_ electrodes can be further improved. As we known, two-dimensional single-layer nanosheets are highly resistant to volume changes, while highly uneven surfaces ensure more active sites (Wang M. et al., 2013; Qi et al., 2018). It was predicted that the self-assembly of 3D hierarchical MoS_2_ nano-flowers with open architecture will maximize the advantages of 2D MoS_2_ nanosheets. Additionally, the conventional chemical exfoliation process will introduce oxygen functional groups to form a single layer of graphene oxide (GO). The loss of π system leads to a poor conductivity of GO. GO have to be reduced to the reduced GO (rGO) to reconstruct the sp2 hybridized network before binding to MoS_2_. Although GO can be partially reduced through high-temperature annealing or chemical reduction, large aggregates are easily formed under van der Waals force. Moreover, MoS_2_ nanostructures tend to separate from the rGO/GO surfaces due to the weak interaction between rGO/GO and MoS_2_. Therefore, the assembly of micron-scale 3D hierarchical MoS_2_ pore nanospheres on the surface of rGO nanosheets was a major challenge.

Herein, inspired by the structure of sandwiches, we constructed a novel 3D flower-like MoS_2_-rGO sandwich nanocomposites (MoS_2_-PrGO) for lithium-ion battery anode through a linking mode and *in-situ* conversion. Supported by the PEO chains, the enlarged interlayer space of rGO not only accommodated as many MoS_2_ nano-flowers as possible, but also acted as a conductive framework. The mild volume expansion of the MoS_2_ nano-flowers during charge-discharge processes was effectively alleviated by the PEO chains. The rGO conductive framework mad all flower-like MoS_2_ stuffings electrochemically active. With this unique design, the 3D flower-like MoS_2_-PrGO sandwich composites exhibited remarkable specific capacity and ultra-stable cyclic performance during the electrochemical measurements.

## Experimental Method

### Preparation of Go

GO was prepared based on improved Hummers method. 1.5 g of potash nitrate (NaNO_3_) and 3.0 g of flake graphite were dissolved in 100 mL of sulfuric acid (H_2_SO_4_, 98%), and stirred for 0.5 h under 10°C. Then, 10.0 g of potassium permanganate (KMnO_4_) was slowly added to the mixture, followed by continuous magnetic stirring for 0.5 h. This mixture was diluted with 700 mL deionized water and continued to stir at 95°C for 1 h. Afterward, 30% hydrogen peroxide (H_2_O_2_, 15 mL) was dropped to the mixture and turned dark green solution into a bright yellow one. The suspension was then washed with 1:10 HCl solution and deionized water for several times. Finally, the obtained GO was freeze-dried for 48 h.

### Preparation of MoS_2_-PrGO

MoS_2_@rGO was synthesized through one step hydrothermal method. Forty milligrams of rGO and 6.9 mg of PEO (Mw = 100,000) were dissolved in 60 ml of deionized water and homogenized by stirring for 12 h. After the addition of 200 mg of Sodium Molybdate Dihydrate (Na_2_MoO_4_•2H_2_O) and 500 mg of Sulfourea (NH_2_CSNH_2_), the mixture was stirred for 0.5 h and poured into 100 ml reactor to reacted at 210°C for 24 h. Naturally cooling to room temperature, the obtained products were washed with deionized water and ethanol for several times. The MoS_2_/rGO nanocomposites were harvested after drying in vacuum at 60°C for 48 h.

### Structure and Morphology Characterization

The phase purity of the synthesized products was examined by X-ray powder diffraction (XRD, Panalytical X' Pert, Holland) by using Cu-Ka radiation (λ = 1.5418 Å). The morphology of the products was characterized by field emission scanning electron microscopy (SEM, Model: JEOL JSM−7000F, Japan) and high-resolution transmission electron microscopy (HRTEM, Model: FEI Titan X 60–300, USA). X-ray photoelectron spectroscopy (XPS) measurements were performed on a Perkin-Elmer model PHI 5600 XPS system with a monochromated aluminum anode as the X-ray source.

### Electrochemical Measurements

Electrochemical tests were carried out in CR 2032 coin cells with lithium metal as the reference and counter electrode. Seventy percent active materials, 20% carbon black and 10 % PVDF were evenly mixed in N-methyl-2-pyrrolidone to form the cathode slurry. The active material loading was controlled at about 2 mg cm^−2^. 1 M LiPF_6_ in ethylene carbonate and diethyl carbonate (V_eth_: V_die_ 1:1) was used as electrolyte. Galvanostatic charge-discharge tests were performed by using a multichannel battery tester (Neware BTS-610) in a voltage window of 0–3.0 V. Cyclic voltammetry curves (CV) were carried out on a PARSTAT 4,000 electrochemical workstation at a scan rate of 0.2 mV s^−1^ in the range of 3.0–0.01 V(vs. Li/Li^+^). Electrochemical impedance spectroscopy (EIS) was tested in the frequency range of 100 kHz−0.01 Hz.

## Results and Discussion

### Synthesis and Characterization

**Figure 1:**
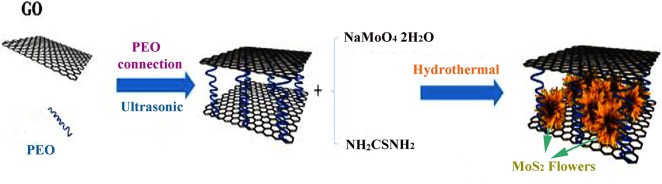
Schematic illustration of the formation process of 3D flower-like MoS_2_-PrGO sandwich composites.

Figure 1 illustrates the preparation mechanism of the 3D flower-like MoS_2_-PrGO nanocomposites. Firstly, under the ultrasonic induction, PEO chains were tightly bound to the GO surface to form a large area of 3D PGO layered spatial structure due to the strong affinity between PEO's non-shared ether-oxygen electrons and GO hydrogen bonds. Secondly, under the electrostatic force, ${\text{MoO}}_{4}^{2-}$ anions approached the GO surface and were mainly captured by the carboxyl, hydroxyl and epoxy groups (Chang and Chen, 2011). Finally, H_2_S released by sulfourea converted ${\text{MoO}}_{4}^{2-}$ and GO *in situ* to MoS_2_ nano-flowers and rGO in the solvothermal process. Therefore, 3D flower-like MoS_2_-PrGO sandwich nanocomposites were successfully obtained.

**Figure 2:**
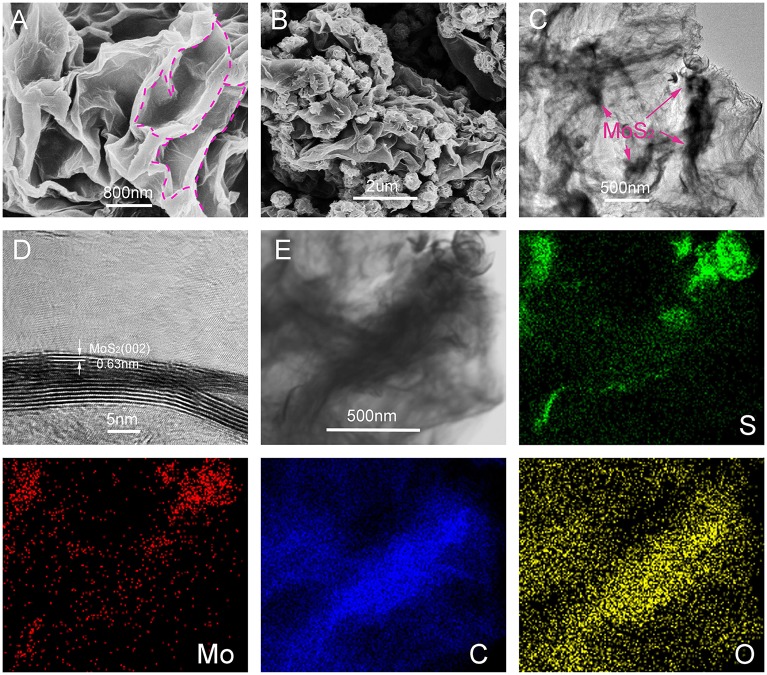
SEM and TEM images of 3D flower-like MoS_2_-PrGO sandwich composites, **(A)** SEM image of GO, **(B)** SEM image of 3D flower-like MoS_2_-PrGO sandwich composites, **(C,D)** TEM and HRTEM images of 3D flower-like MoS_2_-PrGO sandwich composites, **(E)** Bright-field TEM image and corresponding EDX elemental mapping images of 3D flower-like MoS_2_-PrGO sandwich composites.

SEM and TEM were used to describe the morphology of the obtained sandwich nanocomposites. As shown in Figure 2A, the separated 2D ultrathin rGO nano-sheets were assembled into a remarkable 3D layered spatial structure under the linking of PEO. Supported by the PEO chains, the enlarged interlayer space can accommodate a significant number of micron-sized MoS_2_ nano-flowers. Figure 2B shows the morphology of the 3D MoS_2_-PrGO sandwich nanocomposites. The ultrathin MoS_2_ nanosheets were assembled into distinct nano-flowers anchored on the surfaces of rGO hosts. The average size of MoS_2_ nano-flowers was about 200 nm. The results of thermogravimetric analysis (TGA) indicates that the contents of MoS_2_ in the obtained nanocomposites reached 83.3% (Figure S1 and Table S1). This 3D layered sandwich structure is conducive to improve the electronic and ionic conductivity. In addition, the PEO chains buffered the volume expansion of the MoS_2_ nano-flowers during charge/discharge processes. The TEM image of the 3D layered MoS_2_-PrGO sandwich nanocomposites (Figure 2C) reveals that the MoS_2_ nano-flowers were well-encased in the rGO interlayers without aggregation, which is consistent with SEM image. The further HR-TEM image (Figure 2D) displays that the MoS_2_ petals consisted of 5–10 monolayers. The interlayer distance between each monolayer was about 0.62 nm, corresponding to the (002) lattice planes of MoS_2_ (Lin et al., 2017). The corresponding EDX mapping of C, O, S, and Mo reveals that the C and O elements were homogeneously distributed in the specimens (Figure 2E), while the S and Mo elements were agglomerated with remarkable flower-liked hot spots. This further confirmed that the ultrathin MoS_2_ nanosheets were assembled into nano-flowers anchored on the surfaces of rGO hosts, successfully forming a 3D flower-like MoS_2_-PrGO sandwich architecture.

**Figure 3:**
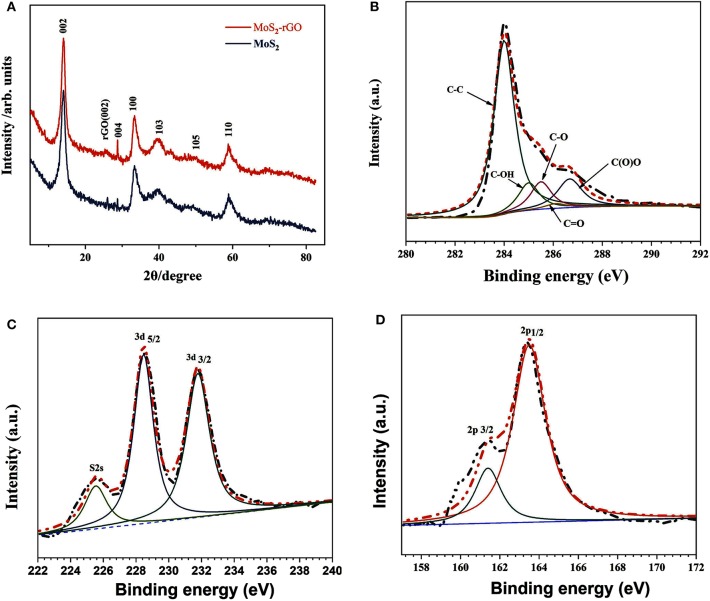
**(A)** XRD patterns of MoS_2_ and 3D flower-like MoS_2_-PrGO sandwich composites, High-resolution XPS spectra of **(B)** C 1 s, **(C)** Mo 3d, and **(D)** S 2p.

Figure 3A presents the XRD pattern of the 3D flower-like MoS_2_-PrGO sandwich composites and the commercial MoS_2_ specimens. All diffraction peaks of the MoS_2_ matched well the diffraction peaks of the typical hexagonal MoS_2_ (JCPDS 37-1492). The obtained hexagonal MoS_2_ had lattice parameters of *a* = *b* = 3.16 Å and *c* = 12.31 Å, which were consisted with the experimental values (Guo et al., 2017). Other than diffraction peaks of the hexagonal MoS_2_, a weak and broad peak appeared in the XRD pattern of the 3D flower-like composites. This additional peak located at 24.5° was attributed to the (002) planes of rGO hosts (He et al., 2014; Chong et al., 2017). The chemical bonding conditions of the 3D flower-like composites were further analyzed by XPS. As shown in Figure 3B, XPS spectrum of the C 1 s core-level was divided into two deconvolution peaks. The intense peak located at around 284.6 eV matched the C–C bonds of rGO nanosheets, and the weak peak near 286.0 eV was contributed by C–O bond (Yang et al., 2009). Figures 3C,D show the XPS spectrums of Mo 3d and S 2p, respectively. The bonding peaks centered at 228.8 and 232.1 eV reflected the bonding states of Mo 3d_5/2_ and Mo 3d_3/2_, implying a standard Mo^4+^ state. The main and satellite peaks of S 2p spectrum were, respectively, located at 161.7 and 162.8 eV with a 1.1 eV energy separation, corresponding to the S^2−^ state (Khawula et al., 2016). The XPS analysis indicated that S and Mo elements in the final products existed in the form of MoS_2_.

### Electrochemical Properties

**Figure 4:**
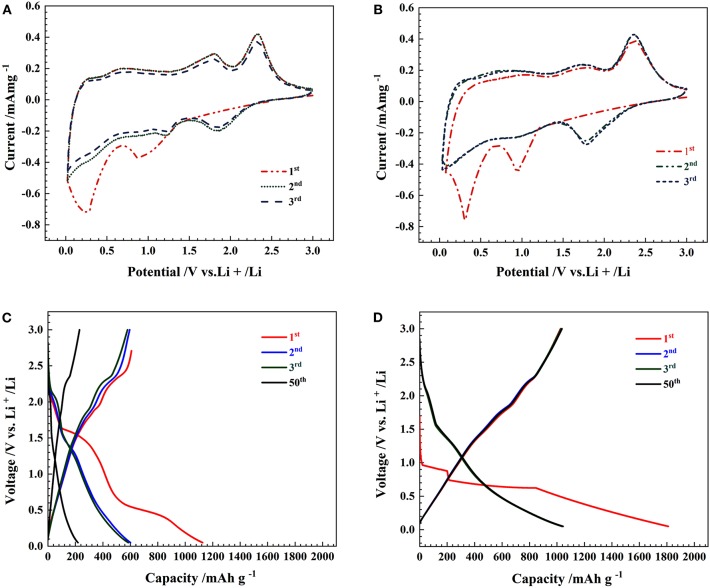
CV curves of **(A)** MoS_2_ and **(B)** 3D flower-like MoS_2_-PrGO sandwich composites, Charge-discharge voltage profiles of **(C)** MoS_2_, and **(D)** 3D flower-like MoS_2_-PrGO sandwich composites.

Figures 4A,B illustrate the first three cyclic CV curves of the commercial MoS_2_ specimens and the 3D flower-like MoS_2_-PrGO sandwich composites. In the first cathodic scan, the CV curves of the commercial MoS_2_ specimens and the 3D flower-like composites exhibited two significant reduction peaks at ~0.88 and 0.26 V. The reduction peak at 0.88 V was derived from the formation of Li_x_MoS_2_ after lithium ions were inserted into (002) plane of MoS_2_ (Liu et al., 2015), while the other peak at 0.26 V was induced by the conversion of MoS_2_ into Mo and Li_2_S. The anodic scan peak around 2.39 V reflected the reaction of Li_2_S into Li^+^ and S. Over the next two cycles, both CV curves displayed an additional reduction peak near 1.86 V. This additional reduction peak was mainly attributed to the formation of gelatinous polymeric layers (Du et al., 2010). The electrochemical reaction mechanism of each peak was written as (Wang Z. et al., 2013).

(1) $$\begin{array}{lll} 2\text{L}{\text{i}}^{+}+\text{S}+2{\text{e}}^{-} & \to & \text{L}{\text{i}}_{2}\text{S} \end{array}$$

(2) $$\begin{array}{lll} \text{Mo}{\text{S}}_{2}+\text{xL}{\text{i}}^{+}+\text{x}{\text{e}}^{-} & \to & \text{L}{\text{i}}_{\text{x}}\text{Mo}{\text{S}}_{2} \end{array}$$

(3) $$\begin{array}{lll} \text{L}{\text{i}}_{\text{x}}\text{Mo}{\text{S}}_{2}+\left(4-\text{x}\right)\text{L}{\text{i}}^{+}+\left(4-\text{x}\right){\text{e}}^{-} & \to & \text{Mo}+2\text{L}{\text{i}}_{2}\text{S} \end{array}$$

Compared with the commercial MoS_2_ specimens, the CV curves of the 3D flower-like composites presented stronger reduction peaks because the electrochemical reaction of lithium with MoS_2_ was synchronous with that of lithium with rGO (Wang and Li, 2007).

Figures 4C,D show the charge-discharge voltage profiles of the commercial MoS_2_ specimens and the 3D flower-like MoS_2_-PrGO sandwich composites at a constant current density of 100 mA g^−1^. During the first discharge, the commercial MoS_2_ anode produced two potential plateaus at 1.4 and 0.5 V. The plateau at 1.4 V corresponded to the formation of Li_x_MoS_2_ lattice and the insertion of lithium ions. The discharge plateau at 0.5 V was attributed to the conversion of MoS_2_ into Mo and Li_2_S, as well as the formation of gelatinous polymeric layers driven by electrochemical degradation of electrolyte (Ding et al., 2011). Consistent with CV test results, the broad plateau at about 2.3 V was a trace of the Li_2_S decomposition. As shown in Figure 4D, although those plateaus recurred in the charge-discharge voltage profiles of the 3D flower-like composites, their width decreased. This indicated that the electron migration rate of the 3D flower-like composites is faster than that of the commercial MoS_2_ specimens. Furthermore, the gentle potential curve slope and large charge capacity indicate that the 3D flower-like composites delivered more reactive sites and nice cycle stability. The first discharge-charge capacities of the 3D flower-like composites were 1,800 and 1,122 mA h g^−1^, respectively, far higher than those of the commercial MoS_2_ specimens. The initial irreversible capacity loss was about 37.7 %, mainly caused by the lithium ion residues in the MoS_2_ lattice and the formation of SEI films (Tang et al., 2009).

**Figure 5:**
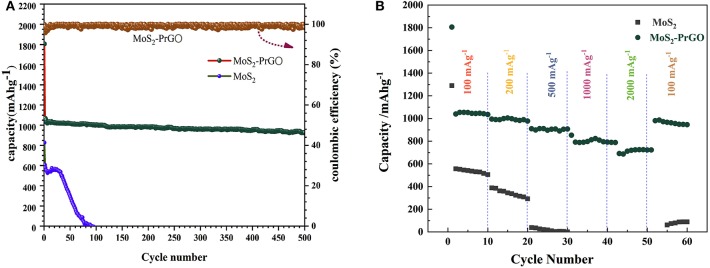
**(A)** Cycling behaviors of MoS_2_ and 3D flower-like MoS_2_-PrGO sandwich composites at a current density of 100 mA g^−1^, **(B)** Rate performances of MoS_2_ and 3D flower-like MoS_2_-PrGO sandwich composites.

Figure 5A plots the cycling performances of the commercial MoS_2_ specimens and the 3D flower-like MoS_2_-PrGO sandwich composites at a constant current density of 100 mA g^−1^. It was obvious that the commercial MoS_2_ electrodes could not afford prolonged cycling and rapidly decayed to zero after 170 cycles, while the 3D flower-like composites exhibited extraordinary cycling behavior and still maintained a reversible capacity of 1,036 mA h g^−1^ after 500 cycles. SEM test results reveals that after 500 cycles, the structure of the 3D flower-like composites was still intact without obvious pulverization (Figure S2). The high reversible capacity and remarkable cycling behavior of the 3D flower-like composites were also reflected in the rate performance. Figure 5B shows the rate cycling behavior of the commercial MoS_2_ specimens and the 3D flower-like MoS_2_-PrGO sandwich composites at various current densities of 100–2,000 mA g^−1^. Compared with the commercial MoS_2_ specimens, the 3D flower-like composites presented an excellent rate performance. Their specific capacity remained at 579 mA h g^−1^ when current density continuously increased to 2,000 mA g^−1^. In contrast, commercial MoS_2_ specimens failed completely before the cycling current increased to 500 mA g^−1^. In addition, when the current density changed to 100 mA g^−1^, the specific capacity of the 3D flower-like composites almost returned to their initial value, and remained stable throughout the subsequent cycling.

**Figure 6:**
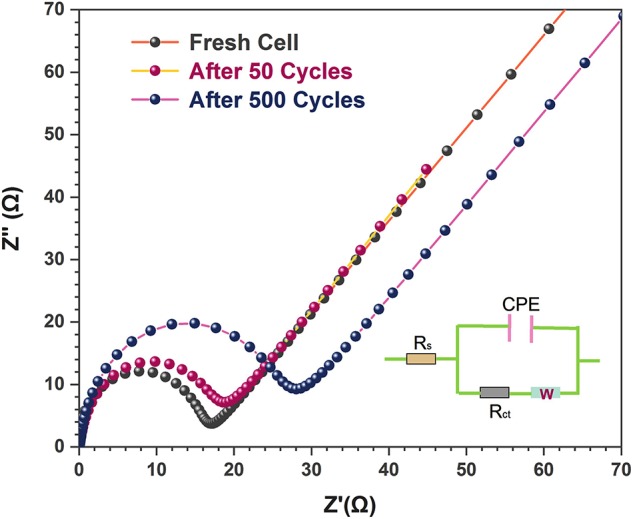
Electrochemical impedance spectra of MoS_2_ and 3D flower-like MoS_2_-PrGO sandwich composites after different cycles.

The excellent electrochemical performance of the 3D flower-like composites was further verified by the EIS measurements. Figure 6 shows the Nyquist plots of the 3D flower-like composite electrodes after different cycles. All Nyquist plots were semi-circular at the high frequencies and oblique in the low frequencies. The semicircle reflected the charge-transfer impedance on electrode (R_ct_), and the slope line in the low-frequency range represented the Warburg impedance (W) associated with the diffusion process of lithium ions (Xu et al., 2013). The Randles equivalent circuit inserted in Figure 6 simulated the corresponding electrochemical system, where CPE represents the double-layer capacitance and Rs is the ohmic resistance. The fresh 3D flower-like composite electrodes exhibited a small semi-circular with a slight increase after 500 cycles. This indicated that the 3D flower-like composite electrodes possessed lower charge-transfer resistance and long-term cycling stability. Their lower charge-transfer resistance is due to the good conductivity of rGO. Furthermore, their unique 3D layered sandwich structure also facilitates rapid transfer of lithium ions upon cycling. Assisted by PEO chains buffering, the robust rGO interlayers effectively suppressed the volume expansion of MoS_2_ and ensured a good cycling performance. In addition, the ultrathin MoS_2_ nanosheets also accommodated a high tolerance to structure changes. Their unique 3D flower-like sandwich structure provided more active sites for the interface reaction between active materials and the lithium-ions, which endowed the final electrodes with a high reversible capacity.

## Conclusions

In summary, the 3D flower-like MoS_2_-PrGO sandwich composites were synthesized by a facile hydrothermal method. PEO with non-shared ether-oxygen electron pairs firstly bridged the separated 2D ultrathin rGO nano-sheets into a remarkable 3D layered spatial structure, and then the MoS_2_ nano-flowers were homogeneously grown on the surfaces of rGO hosts by *in-situ* conversion. This unique 3D flower-like sandwich structure has more active sites and large volume change accommodation. The 3D flower-like composites highlighted high energy density and remarkable cycling stability, and excellent conductivity. Their discharge capacity was retained at 1036 mA h g^−1^ even after 500 cycles at current densities of 100 mA g^−1^. This novel structure design of anode material provides new ideas for improving the electrochemical performances of other metal sulfides.

## Data Availability Statement

All datasets generated for this study are included in the article/Supplementary Material.

## Author Contributions

YZha conducted the experiments. ZZ was the supervisor of this research work. YZha, HZ, ZZ, and SX helped with writing. YZha, YZho, and YW performed the characterization and data analysis. All authors involved the analysis of experimental data and manuscript preparation.

### Conflict of Interest

The authors declare that the research was conducted in the absence of any commercial or financial relationships that could be construed as a potential conflict of interest.
