# Supplementary material for: Three-Dimensional PrGO-Based Sandwich Composites With MoS2 Flowers as Stuffings for Superior Lithium Storage
Source: Front Chem. 2020 Feb 28;8:94. doi: 10.3389/fchem.2020.00094 (PMC7058583; doi:10.3389/fchem.2020.00094)
Supplement: Supplementary file 1 [file Data_Sheet_1.docx]

**Supporting Information**

**Three-Dimensional PrGO-based sandwich composites with MoS_2_ flowers as stuffings for superior lithium storage**

Yangqiang Zhao^1^, Ziying Zhang^1*^, Huizhen Zhang^2^, Yangyang Zhou^1^, Ying Weng^1^, Shisheng Xiong^*3^

^1^School of Materials Engineering, Shanghai University of Engineering Science, Shanghai 201620, China

^2^School of Management, University of Shanghai for Science and Technology, Shanghai 200093, China

^3^School of Information Science and Engineering, Fudan University, Shanghai 200433, China

*Corresponding Authors: Ziying Zhang, Shisheng Xiong

E-mail address: zzying@sues.edu.cn, sxiong@fudan.edu.cn

**
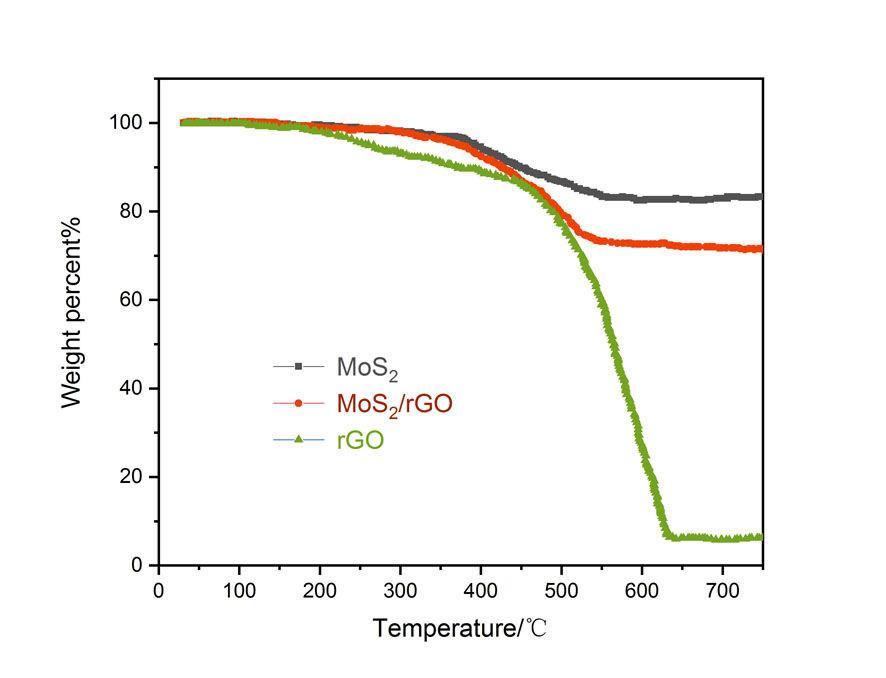
**

Figure S1- TGA curves of MoS_2_, MoS_2_/rGO, and rGO

Table S1. The weight percent of MoS_2_, rGO and MoS_2_/RGO calculated by data in TGA.

| Samples | Weight percent (%) | MoS_2_ (wt%) | rGO (wt%) |
| --- | --- | --- | --- |
| MoS_2_ | 84.6 |  |  |
| rGO | 5.6 |  |  |
| MoS_2_/rGO | 71.3 | 83.3 | 16.7 |


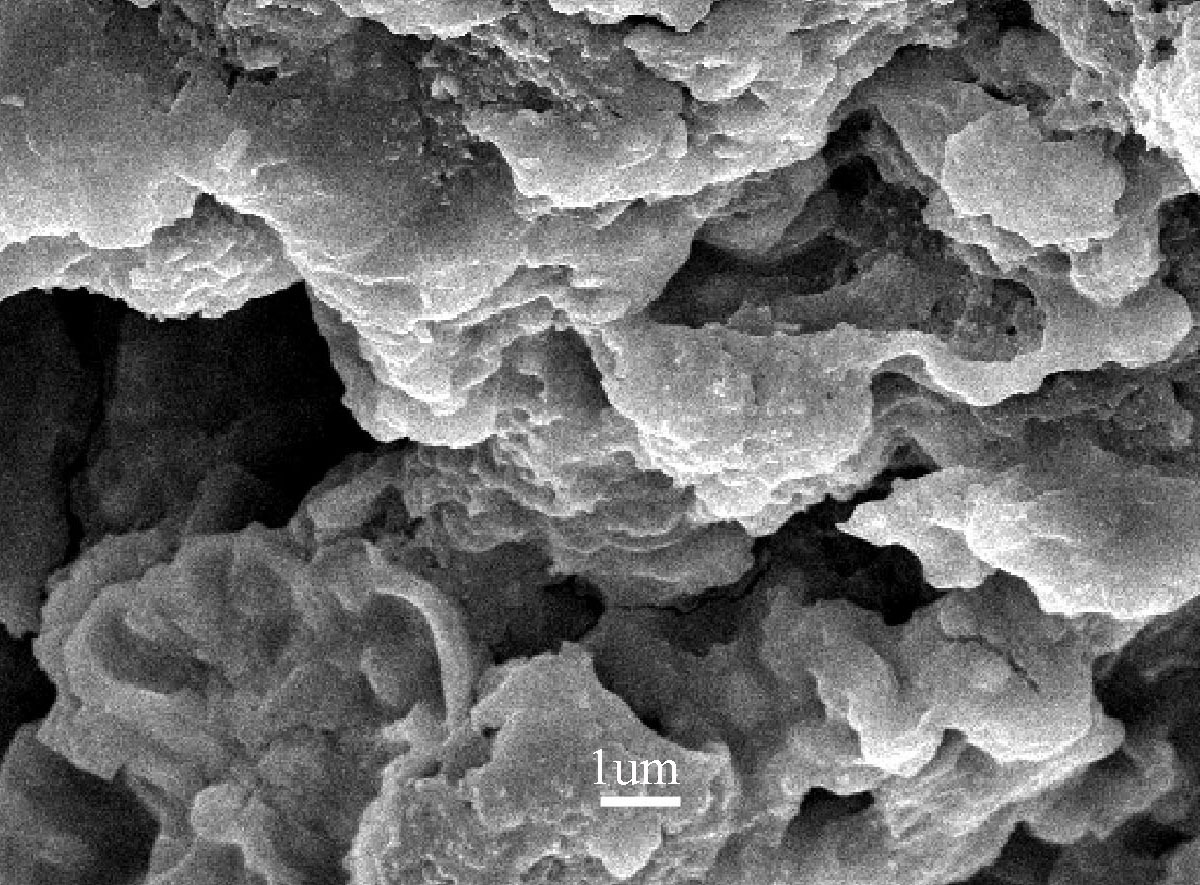


Figure S2- SEM image of MoS_2_/rGO after 500 cycles.
